# Effects of Food Processing on In Vivo Antioxidant and Hepatoprotective Properties of Green Tea Extracts

**DOI:** 10.3390/antiox8120572

**Published:** 2019-11-21

**Authors:** Xiao-Yu Xu, Jie Zheng, Jin-Ming Meng, Ren-You Gan, Qian-Qian Mao, Ao Shang, Bang-Yan Li, Xin-Lin Wei, Hua-Bin Li

**Affiliations:** 1 Guangdong Provincial Key Laboratory of Food, Nutrition and Health, Department of Nutrition, School of Public Health, Sun Yat-Sen University, Guangzhou 510080, China; xuxy53@mail2.sysu.edu.cn (X.-Y.X.); zhengj37@mail2.sysu.edu.cn (J.Z.); mengjm@mail2.sysu.edu.cn (J.-M.M.); maoqq@mail2.sysu.edu.cn (Q.-Q.M.); shangao@mail2.sysu.edu.cn (A.S.); liby35@mail2.sysu.edu.cn (B.-Y.L.); 2 Institute of Urban Agriculture, Chinese Academy of Agricultural Sciences, Chengdu 610213, China; 3 Department of Food Science & Technology, School of Agriculture and Biology, Shanghai Jiao Tong University, Shanghai 200240, China; weixinlin@sjtu.edu.cn

**Keywords:** green tea extract, food processing, tannase, ultrasound, antioxidant activity, liver injury

## Abstract

Food processing can affect the nutrition and safety of foods. A previous study showed that tannase and ultrasound treatment could significantly increase the antioxidant activities of green tea extracts according to in vitro evaluation methods. Since the results from in vitro and in vivo experiments may be inconsistent, the in vivo antioxidant activities of the extracts were studied using a mouse model of alcohol-induced acute liver injury in this study. Results showed that all the extracts decreased the levels of aspartate transaminase and alanine aminotransferase in serum, reduced the levels of malondialdehyde and triacylglycerol in the liver, and increased the levels of catalase and glutathione in the liver, which can alleviate hepatic oxidative injury. In addition, the differences between treated and original extracts were not significant in vivo. In some cases, the food processing can have a negative effect on in vivo antioxidant activities. That is, although tannase and ultrasound treatment can significantly increase the antioxidant activities of green tea extracts in vitro, it cannot improve the in vivo antioxidant activities, which indicates that some food processing might not always have positive effects on products for human benefits.

## 1. Introduction

The antioxidant properties of food include the capacities of reducing, scavenging radicals, chelating metal ions, inhibiting oxidative enzymes, and activities as antioxidative enzymes [1,2,3,4,5,6]. Many methods have been developed for the evaluation of in vitro antioxidant activities of natural products, and some of them showed strong antioxidant activities, such as vegetables, fruits, cereals, algae, and tea [7,8,9,10,11,12,13,14,15,16].

Oxidative stress can be caused in the human body due to the overproduction of reactive oxygen species (ROS) over the capability of cells to present an effective antioxidant response [17,18]. The oxidative stress results in cellular dysfunction and is involved in various chronic disease initiation and progression, such as diabetes, cancer, neurodegeneration, aging, cardiovascular diseases, and liver diseases [19,20,21]. Due to strong in vitro antioxidant activities, some natural products have been regarded as effective agents for the prevention and management of several chronic diseases [22,23,24,25]. On the other hand, the formation of ROS in vivo can be stimulated due to alcohol metabolism [26,27]. The animal model with acute alcohol administration has been used to investigate the in vivo antioxidant activities of food [28,29], and it often occurs accompanied by liver injury, which can be used for hepatoprotection studies [30]. Hence, we used an animal model with acute alcohol-induced liver injury to evaluate the in vivo antioxidant and hepatoprotective activities of green tea extracts with different processing in this study.

Green tea (*Camellia sinensis L.*) has been reported to show multiple bioactivities with health benefits, such as antioxidant, anti-inflammation, hepatoprotection, cardiovascular protection, neuroprotection, and anti-cancer [31,32,33,34,35,36]. The epidemiological studies showed that green tea consumption can result in a decreased risk of metabolic syndrome, but there is not enough evidence to draw a strong conclusion regarding tea and non-alcoholic fatty liver [37]. Moreover, accumulating in vivo evidence suggested that green tea showed hepatoprotective effects, which can ameliorate the liver injury induced by alcohol, cholesterol, chemicals, or drugs [38,39,40,41]. These benefits are mainly due to the richness of bioactive compounds like polyphenols, polysaccharides, and amino acids [42,43,44,45]. The major polyphenols in green tea include catechins and phenolic acids [46]. Catechins are mainly composed of (−)-epicatechin (EC), (−)-epigallocatechin (EGC), (−)-epicatechin gallate (ECG), and (−)-epigallocatechin gallate (EGCG), and phenolic acids include gallic, coumaric, caffeic acids, etc. [47,48]. Furthermore, many findings have demonstrated that the catechins and phenolic acids are responsible for the antioxidant properties of green tea, which has protective effects against many diseases, such as diabetes, cancer, hypertension, and cardiovascular diseases [49,50,51,52,53]. However, there is potential hepatotoxicity induced by the overdose of EGCG [54,55]. Hence, food processing, such as enzymatic treatment, is used to reduce the content of EGCG in green tea extracts to eliminate its negative effects [56]. 

Numerous types of enzymes are currently used in food processing to meet the demands of a broad variety of food products [57,58]. Additionally, the use of enzymes in foods produces other substances from enzymatic hydrolysis and improves the quality of food products [59,60,61,62]. Due to its capacities in catalyzing hydrolysis of gallic acid esters and hydrolysable tannins, tannase (tannin acyl hydrolase EC 3.1.1.20) is widely used in the production of gallic acid [63,64]. On the other hand, ultrasound has been extensively used in food processing, improving the quality and safety of products [62,65,66]. Also, ultrasound creates cavitation and promotes heat and mass transfer, which accelerates chemical reactions, such as enzymatic reactions [67,68,69]. However, some compounds in products have been changed during food processing, and they might pose negative effects on the quality and health benefits of foods [70]. Most present studies use in vitro methods to evaluate antioxidant properties of food after they are treated with different processing methods. But fewer studies were found about the in vivo antioxidant activities of processed and original products. Our previous study revealed that tannase and ultrasound treatments markedly increase the antioxidant activities of green tea extracts based on the results of in vitro assays. In this study, we aim to investigate the in vivo effects of green tea extracts processed by tannase and ultrasound against oxidative stress and liver injury induced by alcohol.

## 2. Materials and Methods

### 2.1. Chemicals and Reagents

Tannase (200 U/g) was bought from Yuanye Biological Technology Co., Ltd. (Shanghai, China). All the other chemicals or reagents were of analytical grade. The kits of aspartate transaminase (AST), alanine aminotransferase (ALT), triglyceride (TG), malondialdehyde (MDA), glutathione (GSH), superoxide dismutase (SOD), catalase (CAT), and total protein were purchased from Nanjing Jiancheng Bioengineering Institute (Nanjing, China). The deionized water was used for all experiments.

### 2.2. Preparation of Green Tea Extracts

Green tea was purchased from the local market of Guangzhou, China, and was ground into powder which was filtered through a 100 mesh sieve. The deionized water was used to mix with the powder (50 g/L, *w*/*v*), and then the mixture was heated at 85 °C for 30 min in a water bath and centrifugated at 4200× *g* for 30 min. The supernatants were collected as the green tea extracts for further experiments.

According to the previous study, the green tea extracts showed the highest antioxidant activities in vitro under the optimal extraction conditions with 0.1 M citrate-phosphate buffer (pH 4.62), ultrasonic temperature of 44.12 °C, ultrasonic time of 12.17 min, tannase concentration of 1 mg/mL, and ultrasonic power of 360 W [71]. The green tea extracts were divided into four groups with different treatments. For the first group, the treatment of ultrasound and tannase (UST) was conducted by mixing the green tea extract with 1 mg/mL tannase in 0.1 M citrate-phosphate buffer (pH 4.62) and using an ultrasonic device (Kejin Ultrasonic Equipment Factory, Guangzhou, China) for 12.17 min at 44.12 °C under 360 W. For the second group, the ultrasound treatment (US) was carried out by mixing the extract with 0.1 M citrate-phosphate buffer (pH 4.62) without tannase and treating with ultrasound for 12.17 min at 44.12 °C under 360 W. For the next group, the only tannase (TAN) treatment was mixed with 1 mg/mL tannase in 0.1 M citrate-phosphate buffer (pH 4.62), and placed in a water bath at 44.12 °C for 12.17 min. The group with the green tea extract (GTE) had a treatment which included the dilution of original extract with the same buffer solution. After the completion of treatment, the mixtures were fully mingled by a vortexing machine. Then they were placed in the water bath at 100 °C for 10 min to inactivate tannase and cooled down to room temperature. The mixture was centrifugated at 4200× *g* for 10 min, and the supernatant was collected for further experiments.

The extracts from UST, US, TAN, and GTE groups were later dried via the vacuum rotary evaporator. The dried crude extracts were collected and dissolved in deionized water for the animal study.

### 2.3. Animal Study

Male Kunming mice (20–25 g) were obtained from the Experimental Animal Center of Sun Yat-Sen University, Guangzhou, China. All procedures were strictly carried out according to the principles of “laboratory animal care and use” approved by School of Public Health, Sun Yat-Sen University (No. 2019-002; 28 February 2019). The mice were fed in a specific pathogen free (SPF) animal room under a temperature of 22 ± 0.5 °C, relative humidity of 40–60%, and 12 h light/dark cycle. After the mice had acclimated for one week, they were randomly divided into different groups (6 mice in each group), including control, model, and treatment groups. The treatment groups were fed intragastrically with the solutions of US (50 mg/kg body weight), TAN (50 mg/kg body weight), GTE (50 mg/kg body weight), and UST (50, 100, 200 mg/kg body weight) for 7 days. The model and control groups received the deionized water. On the seventh day, all treatment and model groups were fed intragastrically with 52% alcohol (*v*/*v*, 10 mL/kg body weight) 30 min after the last administration, while the control group received the deionized water. After 6 h fasting following the last administration of alcohol, all mice were weighed and anaesthetized to sacrifice. Then the blood samples were collected and centrifuged at 3000× *g* for 10 min. The serum was isolated for AST and ALT evaluation which followed the instructions of the commercial kits. The liver was harvested and weighed. In order to control potential oxidation of the sample, the low temperature condition was adopted. That is, the 10% (*w*/*v*) liver homogenate was prepared by mixing the liver and ice-cold 0.9% normal saline solution in a glass tube that was put in the ice box and the liver was grinded with a glass grinder [29,72,73]. The homogenate of liver was centrifuged at 2500× *g* for 10 min to obtain the supernatant which was used for the biochemical assays.

### 2.4. Biochemical Assays

The determination of SOD, CAT, GSH, MDA, TG, and total protein followed the instructions of the Nanjing Jiancheng commercial kits produced by Nanjing Jiancheng Bioengineering Institute, Nanjing, China [29,72,73,74,75,76]. (1) Determination of SOD activity: the xanthine and xanthine oxidase reacted to produce superoxide radicals. The radicals oxidated hydroxylamine to induce nitrite that reacted with a color developing agent to produce a purple-red compound. When the sample contained SOD, it reduced the production of nitrite, which was reflected on a decrease in absorbance. The liver homogenate was diluted by ice-cold saline solution to 0.25% (*w*/*v*), and the 50 μL was mixed with reagents. The mixture was placed in room temperature for 10 min. The absorbance was detected at 550 nm using a spectrophotometer. (2) Determination of CAT activity: CAT catalyzed the H_2_O_2_ decomposition, and the remaining H_2_O_2_ reacted with ammonium molybdate to produce a light yellow compound. The activity of CAT was calculated based on the change in absorbance. The liver homogenate was diluted by ice-cold saline solution to 0.5% (*w*/*v*), and the 50 μL was mixed with reagents. The absorbance of mixture was detected at 405 nm using the microplate reader. (3) Determination of GSH content: The reaction of GSH and 5,5′-dithiobis-(2-nitrobenzoic acid) (DTNB) produced a yellow compound. The GSH content was determined by the colorimetry. The 100 μL liver homogenate (10%, *w*/*v*) was mixed with 0.1 mL precipitant, and the mixture was centrifuged at 3500× *g* for 20 min to obtain the supernatant. The 100 μL supernatant was mixed with reagents, and placed in room temperature for 5 min. The absorbance of mixture was determined at 405 nm using the microplate reader. (4) Determination of MDA content: The reaction of MDA with thiobarbituric acid (TBA) led to a red product that had an absorbance peak at 532 nm. The 100 μL liver homogenate (10%, *w/v*) was mixed with reagents, and put in a water bath at 95 °C for 40 min. After the mixture was cooled down, it was centrifuged at 4000× *g* for 10 min. The absorbance of the supernatant was detected at 532 nm using a spectrophotometer. (5) Determination of TG content: TG was hydrolyzed into glycerol and fatty acids by the lipase. The reaction of glycerol and adenosine triphosphate (ATP) was catalyzed by glycerol kinase (GK) and produced glycerol-3-phosphate, which was further oxidized into H_2_O_2_ and dihydroxyacetone phosphate by glycerophosphate oxidase. H_2_O_2_ reacted with 4-aminoantipyrine (4-AAP) and p-chlorophenol under the catalysis of peroxidase to produce a red quinone compound, and its color was proportional to the TG content. The 2.5 μL liver homogenate (10%, *w*/*v*) was mixed with reagents and placed in the water bath at 37 °C for 10 min. The absorbance of mixture was detected at 510 nm using the microplate reader. (6) Determination of AST activity: AST could act on α-ketoglutaric acid and aspartic acid to produce oxaloacetic acid and glutamic acid. The oxaloacetic acid decarboxylated into pyruvate acid that reacted with 2,4-dinitrophenylhydrazine (DNPH) to produce 2,4-dinitrophenylhydrazone which was a reddish brown compound under alkaline conditions. The 5 μL serum was mixed with the reagents and placed at room temperature for 15 min. The absorbance was detected at 510 nm using a microplate reader. (7) Determination of ALT activity: Under the condition of 37 °C and pH 7.4, ALT acted on alanine and α-ketoglutaric acid to produce pyruvate acid and glutamic acid. After 30 min, DNPH in hydrochloric acid solution was added to form acetone phenylhydrazone that was a reddish brown compound under alkaline conditions. The 5 μL serum was mixed with the reagents and placed at room temperature for 15 min. The absorbance was recorded at 510 nm using the microplate reader. (7) Determination of total protein: The protein reduced Cu^2+^ to Cu^+^ under the alkaline conditions, and Cu^+^ reacted with the bicinchoninic acid (BCA) reagent to form a purple complex compound that had an absorbance peak at 562 nm. The absorbance was proportional to the concentration of total protein. The liver homogenate was diluted by ice-cold saline solution to 0.5% (*w*/*v*), and the 10 μL diluted liver homogenate was mixed with the reagents. The mixture was placed at 37 °C for 30 min and the microplate reader was used to detect the absorbance at 562 nm.

### 2.5. Statistical Analysis

All experiments were conducted independently three times, and the results were presented as mean ± standard deviation (SD). The statistical analysis was performed by using SPSS 19.0 (IBM SPSS Statistics, IBM Corp, Somers, NY, USA). One-way ANOVA plus a post hoc least-significant difference (LSD) test was utilized to analyze the significance of differences for each group, and the statistical significance was defined at *p* < 0.05.

## 3. Results and Discussion

### 3.1. Effects of Extracts on Antioxidant Enzymes, GSH, and MDA in the Liver

**Figure 1:**
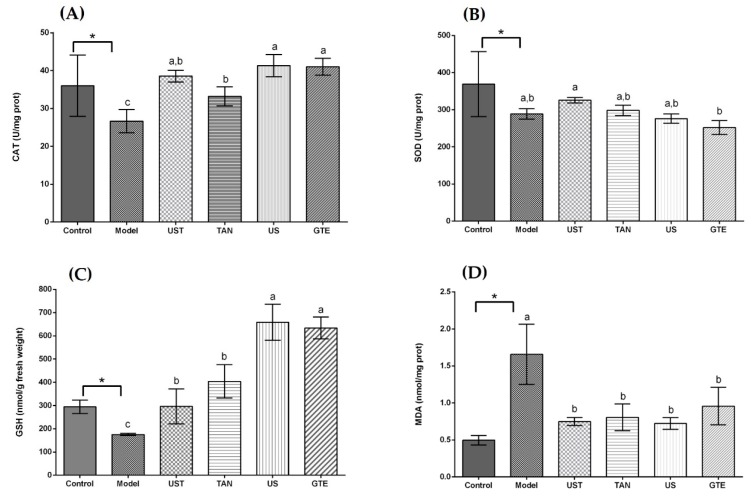
Effects of different extracts on catalase (CAT), superoxide dismutase (SOD), glutathione (GSH), and malondialdehyde (MDA) in the liver. (**A**) CAT, (**B**) SOD, (**C**) GSH, and (**D**) MDA: extracts from different methods (50 mg/kg body weight). One unit of CAT activity is defined as the amount of protein which decomposes 1 μmol H_2_O_2_ per second. One unit for SOD activity is defined as the amount of protein necessary to inhibit 50% of the SOD reaction where superoxide radicals oxidize hydrosylamine to produce nitrite. UST, the group treated with ultrasound and tannase; TAN, the group treated with only tannase; US, the group treated with only ultrasound; green tea extract (GTE), the group treated without ultrasound and tannase. The values are presented as means ± SD. Bars with different letters (a–c) are significantly different (*p* < 0.05). * *p* < 0.05, the model group vs. the control group.

The animal experiments were conducted with the model of alcohol-induced liver injury to assess antioxidant activities of the extracts in vivo. The alcohol administration was observed to induce oxidative stress in mice. Compared with the control group, the model group showed an obvious decrease in CAT and SOD activities as well as GSH content, and an increase in MDA content in the mouse liver (*p* < 0.05, Figure 1).

**Figure 2:**
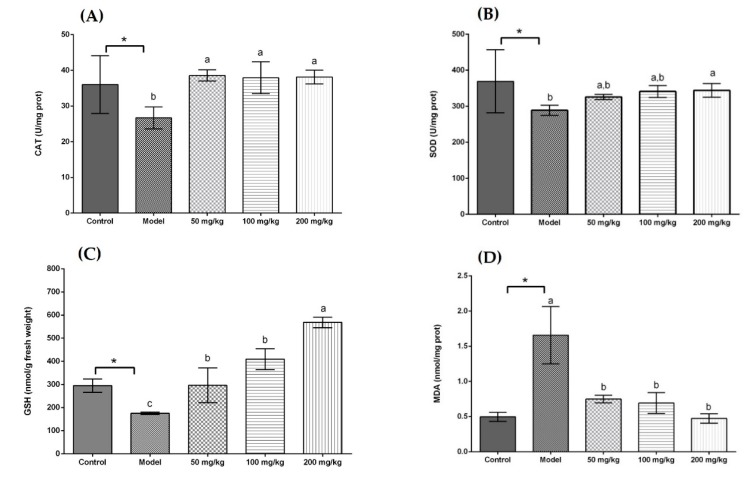
Effects of different doses of UST extracts on CAT, SOD, GSH, and MDA in liver. (**A**) CAT, (**B**) SOD, (**C**) GSH, and (**D**) MDA: UST extract (50, 100, and 200 mg/kg body weight). One unit of CAT activity is defined as the amount of protein which decomposes 1 μmol H_2_O_2_ per second. One unit for SOD activity is defined as the amount of protein necessary to inhibit 50% of the SOD reaction where superoxide radicals oxidize hydrosylamine to produce nitrite. UST, the group treated with ultrasound and tannase; TAN, the group treated with only tannase; US, the group treated with only ultrasound; GTE, the group treated without ultrasound and tannase. The values are presented as means ± SD. Bars with different letters (a–c) are significantly different (*p* < 0.05). * *p* < 0.05, the model group vs. the control group.

As displayed in Figure 1A, all treatment groups at the same dose (50 mg/kg body weight) significantly increased the CAT activity in comparison with the model group. In addition, there was no significant difference in CAT activity among the UST, US, and GTE groups. However, the TAN group showed a negative effect on CAT activity compared with the GTE group. This is because tannase might induce the degradation of some related bioactive compounds [71]. Seen from Figure 1B, although the activity of SOD in the UST group was significantly higher than that of the GTE group, all treatment groups did not increase SOD activity compared with the model group. However, treatment with 200 mg/kg body weight could increase SOD activity (Figure 2B). Therefore, the dose of 50 mg/kg body weight was too low to increase the SOD activity. From Figure 1C, all treatment groups improved GSH content significantly when they were compared with the model group. In addition, there was no marked difference in the levels of GSH between the US and GTE groups. However, the UST and TAN groups showed significantly lower levels of GSH than the GTE groups. These results suggest that tannase treatment might pose negative effects on the in vivo antioxidant activities of green tea extract. As shown in Figure 1D, green tea extracts by different methods reversed the alcohol-induced increase in the level of hepatic MDA, but the differences between groups were not significant. For another thing, the effects of UST at different doses (50, 100, and 200 mg/kg body weight) were shown in Figure 2, and they all increased the activities of CAT and SOD as well as GSH content and lowered the level of MDA. There was no distinct dose-dependent response for the levels of CAT, SOD, and MDA. However, a higher dose showed a stronger effect on GSH content.

Acute alcohol consumption has been reported to induce oxidative stress and stimulate lipid peroxidation, leading to hepatic dysfunction [77]. It produces free radicals and promotes the development of liver diseases. Thus, the activation of antioxidant enzymes for scavenging free radicals is essential for the protection against alcoholic liver disease. SOD and CAT are important antioxidant enzymes in the defence against oxidative damage. SOD acts on removing superoxide, and CAT catalyses the decomposition of hydrogen peroxide [30]. The contents of GSH are also a crucial indicator reflecting the antioxidant and oxidant status in vivo [78]. MDA is an important product of lipid peroxidation, and its content shows the degree of interaction of ROS with polyunsaturated fatty acid [79].

The present study showed that the administration of extracts increased the levels of CAT, SOD, and GSH, and decreased the contents of MDA in the liver as compared to the model group. However, the differences among groups were not significant in the assays of SOD and MDA. On the other hand, the groups with tannase treatment showed low CAT activity and GSH content, indicating that tannase might degrade some other compounds and affect antioxidant activity negatively in vivo. Overall, the tannase and ultrasound treatment had no significant beneficial effects on the antioxidant enzymes and MDA compared with GTE groups, even posing negative effects on the GSH content.

### 3.2. Effects of Extracts on AST and ALT in Serum

**Figure 3:**
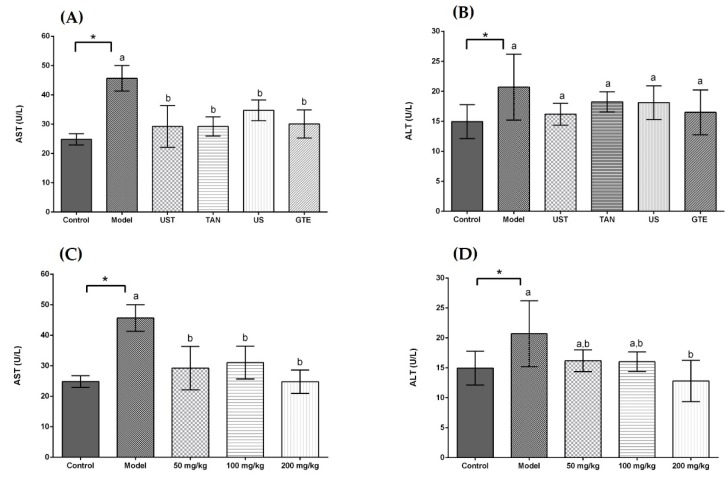
Effects of different extracts on serum aspartate transaminase (AST) and alanine aminotransferase (ALT) activities. (**A**) AST and (**B**) ALT: extracts from different methods (50 mg/kg body weight). (**C**) AST and (**D**) ALT: UST extracts (50, 100, and 200 mg/kg body weight). UST, the group treated with ultrasound and tannase; TAN, the group treated with only tannase; US, the group treated with only ultrasound; GTE, the group treated without ultrasound and tannase. The values are presented as means ± SD. Bars with different letters (a,b) are significantly different (*p* < 0.05). * *p* < 0.05, the model group vs. the control group.

The activities of serum aspartate transaminase (AST) and alanine transaminase (ALT) were measured to investigate the effects of extracts on liver injury induced by acute alcohol intake. In Figure 3, the serum AST and ALT activities were increased in the model group compared with the control group (*p* < 0.05). All treatment groups significantly decreased the serum AST activities in comparison with the model group, but there was no significance among the treatment groups. On the other hand, all treatment groups non-significantly decreased the serum ALT activities compared with the model group. As shown in Figure 3C,D, the ingestion of UST extracts significantly ameliorated the alcohol-induced increase in AST activities, but the dose-dependent effect was not significant. For the ALT activity, the highest dose of UST extracts decreased significantly the activity of ALT in comparison with the model group.

AST and ALT are known as effective markers for liver function. In response to liver damage, AST and ALT are released to plasma from hepatocytes, and the levels of serum AST and ALT are enhanced [80]. In this study, the model groups showed higher serum AST and ALT activities than the control group, which indicated the injury in the liver. The extracts with different methods reduced the serum AST and ALT activities, but the differences among groups were not significant. It indicated that the treatment of ultrasound or tannase contributed little to the reduction in serum ALT and AST activities compared with GTE group. In addition, treatment groups decreased non-significantly the ALT activity in the comparison with the model group. It might be because the doses of extracts were too small to produce a significant effect. In the dose-dependent experiment, a higher dose of UST extract showed a more potent effect on attenuating the abnormal increase in serum ALT activities (Figure 3D). It suggested that using appropriate doses of extracts obtained from the combined treatment of ultrasound and tannase in green tea could diminish the liver dysfunction induced by alcohol in vivo.

### 3.3. Effects of Extracts on TG in Liver

**Figure 4:**
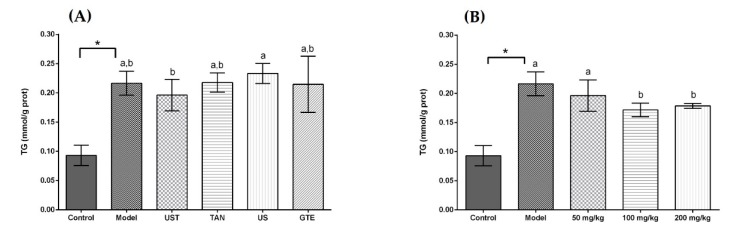
Effects of different extracts on the levels of triglyceride (TG). (**A**) Extracts from different methods (50 mg/kg body weight). (**B**) UST extracts (50, 100, and 200 mg/kg body weight). UST, the group treated with ultrasound and tannase; TAN, the group treated with only tannase; US, the group treated with only ultrasound; GTE, the group treated without ultrasound and tannase. The values are presented as means ± SD. Bars with different letters (a,b) are significantly different (*p* < 0.05). * *p* < 0.05, the model group vs. the control group.

Acute alcohol intake resulted in disturbed lipid metabolism with an increase in hepatic TG. Figure 4 displays the effects of extracts on TG level in liver tissue, and a significant elevation in the level of TG was observed in the model group (*p* < 0.05). The administration of extracts from different methods non-significantly decreased the level of TG in liver tissue compared with the model group. In addition, the results showed that high doses of UST extract could lower the hepatic TG significantly compared with the model group in Figure 4B, but the dose-dependent effect was not obvious.

Excessive drinking can lead to lipid production in the liver, and the accumulation of adipose in liver tissue promotes the progress of relevant diseases [81]. The results revealed that the ultrasound and tannase treatment had little effect in reducing lipid accumulation, and there was no significant difference compared with the GTE group. On the other hand, a high dose of UST extracts could significantly reduce the level of TG in the liver. These results indicated that a certain dose of UST extract could be effective in reducing lipids in the liver.

In our previous study, the treated extracts showed stronger in vitro antioxidant activities than the original extracts, and the contents of several compounds were determined using the HPLC method [71]. HPLC results revealed that tannase and ultrasound treatment increased gallic acid content, while the original extract had a higher EGCG content than the treated extracts. It indicated that the in vitro antioxidant activities of UST and TAN were mainly attributed to the content of gallic acid, while the antioxidant properties of US and GTE mainly depended on the contents of EGCG. On the other hand, this study showed that the differences between treated and original extracts were not significant in vivo, and to our surprise, the food processing even posed a negative effect on in vivo antioxidant activities in some cases. Therefore, the results from in vivo and in vitro studies on antioxidant activities were inconsistent, suggesting that some food processing might not always have positive effects on health benefits. This study also indicated that in the future, the effects of food processing on the quality of products should not be evaluated only using in vitro methods, and in vivo evaluation methods should be adopted.

### 3.4. Histopathological Observation

**Figure 5:**
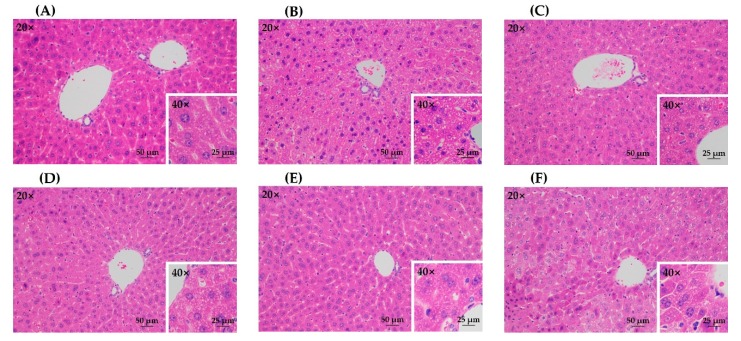
The histopathological observation of hematoxylin and eosin (H&E)-stained liver tissue slices. (**A**) control group; (**B**) model group; (**C**) UST group (50 mg/kg body weight); (**D**) TAN group (50 mg/kg body weight); (**E**) US group (50 mg/kg body weight); (**F**) GTE group (50 mg/kg body weight). UST, the group treated with ultrasound and tannase; TAN, the group treated with only tannase; US, the group treated with only ultrasound; GTE, the group treated without ultrasound and tannase.

The histopathological analysis on hematoxylin and eosin (H&E)-stained liver tissue slices further confirmed the protective effects of all treatment groups against acute alcohol liver injury (Figure 5). The model group showed obvious pathologic changes such as disordered cell arrangement and lipid droplets accumulation, while the control group had no significant damage (Figure 5A,B). All treatment groups presented less steatosis than the model group (Figure 5C–F), which indicated that the lesion induced by acute alcohol administration was attenuated by green tea extracts from different treatment methods. In addition, there was no obvious difference among different treatment groups.

## 4. Conclusions

This study investigated the effects of food processing (tannase and ultrasound treatments) on in vivo antioxidant and hepatoprotective properties of green tea extracts. Results showed that green tea extracts with ultrasound and tannase treatment could attenuate the oxidative stress induced by acute alcohol administration. It increased the activities of antioxidant enzymes, such as SOD and CAT, and the content of antioxidants such as GSH, and reduced the level of MDA. However, there was no significant difference between the treated and original extracts. To our surprise, the tannase treatment even had negative effects on the in vivo antioxidant activities of extracts, which might be related to its degradation of some compounds. In addition, the dose-effect relationship was not significant in green tea extracts with tannase and ultrasound treatment. Thus, it was indicated that the in vitro and in vivo antioxidant activities could be inconsistent, which might be affected by many other factors, such as metabolism and bioavailability. Moreover, the effects of food processing on properties of products should not be evaluated only using in vitro methods, and more in vivo evaluation methods should be carried out. Also, further studies are needed on the necessity of using different food processing methods to produce functional compounds.
